# OXPHOS complex deficiency in congenital myopathy: A systematic review

**DOI:** 10.1111/eci.70114

**Published:** 2025-09-11

**Authors:** Megan J. du Preez, Maryke Schoonen, Monray E. Williams, Michelle Bisschoff, Francois H. van der Westhuizen

**Affiliations:** ^1^ Mitochondria Research Group, Biomedical and Molecular Metabolism Research (BioMMet) North‐West University Potchefstroom South Africa

**Keywords:** congenital myopathy, good health and well‐being, mitochondrial disease, mitochondrial dysfunction, neuromuscular diseases diagnosis, oxidative phosphorylation

## Abstract

**Background:**

Congenital myopathies are inherited neuromuscular disorders characterized by early‐onset muscle weakness and distinct histopathological features. Although mitochondrial involvement in congenital myopathy is well recognized in its pathophysiology, oxidative phosphorylation (OXPHOS) complex dysfunction, which is associated with primary mitochondrial diseases (MD), is not. This systematic review aimed to evaluate the prevalence and characteristics of reported OXPHOS complex dysfunction in genetically confirmed congenital myopathy cases.

**Methods:**

A systematic literature search was conducted in PubMed, Scopus and Web of Science. The search strategy was developed according to PRISMA guidelines. Two independent reviewers screened the studies for inclusion. Eligible studies reported genetically confirmed congenital myopathy cases or disease models and included diagnostic OXPHOS complex analyses via enzyme kinetic assays and/or protein/RNA expression.

**Results:**

Of 5841 studies screened, 23 publications (2009–2025) met the inclusion criteria, comprising 45 congenital myopathy cases. OXPHOS complex dysfunction was reported in 78% of these cases, including all human cases where OXPHOS enzymology was performed. Nine congenital myopathy‐associated genes were involved in the cases, with *RYR1* being the most frequent. No definitive genotype–phenotype relationship was established between specific genes and affected complexes.

**Conclusions:**

OXPHOS complex dysfunction in congenital myopathy appears to be more prevalent than previously recognized, challenging the traditional view that associates such dysfunction exclusively with MD. This emerging evidence suggests that mitochondrial involvement in congenital myopathy is not incidental but may represent a meaningful aspect of its pathophysiology. The potential dysregulation of OXPHOS in congenital myopathy has implications for refining diagnostic frameworks for both congenital myopathy and MD.

## Graphical abstract

This systematic review assessed oxidative phosphorylation (OXPHOS) complex dysfunction in genetically confirmed congenital myopathies (CM). Among 5841 studies screened, 23 publications, comprising 45 CM cases, met the inclusion criteria. OXPHOS dysfunction was identified in 78% of cases, particularly where enzymology was performed, with *RYR1* most frequently implicated. No clear genotype–phenotype correlation emerged. Findings challenge the notion that OXPHOS dysfunction is exclusive to mitochondrial diseases (MD), suggesting it plays a significant role in CM pathophysiology and may impact diagnostic strategies for CM and MD.

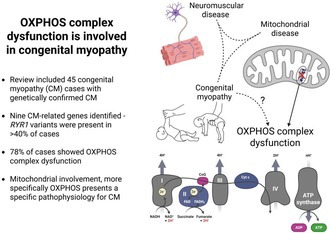

## 1 INTRODUCTION

Congenital myopathies are a heterogeneous group of inherited neuromuscular disorders characterized by early‐onset muscle weakness, hypotonia and specific histopathological features on muscle biopsy.^1^ Genetic variants in a wide range of known causative genes, along with diverse inheritance patterns, contribute to a significant overlap between the genotype and congenital myopathy phenotype.^2^, ^3^ While traditionally classified based on histological and genetic findings, growing evidence suggests that mitochondrial energy metabolism is frequently disrupted in congenital myopathies, leading to secondary mitochondrial dysfunction and other functional impairments in muscle tissue.^4^ There is a well‐documented overlap between congenital myopathies and primary mitochondrial disease (MD), which is a distinct group of neuromuscular diseases resulting from mutations in genes encoding the structural components of, or machinery required for, oxidative phosphorylation (OXPHOS).^5^, ^6^ MD can occur at any stage of life; it is the most common group of inherited metabolic disorders and is the result of mutations in more than 350 genes from both the nuclear and mitochondrial genomes.^7^, ^8^, ^9^ Like congenital myopathies, MD also presents with heterogeneous clinical phenotypes involving energy‐demanding tissues, but with the additional diagnostic characteristic of having a structural and functional deficiency of one or a combination of OXPHOS enzymes. Kinetic assays and enzyme histochemistry of the individual enzymes in muscle or skin fibroblasts, integrated with genomic and clinical data, have been common practice to provide a precise diagnosis for MD in a clinical setting.^8^, ^9^, ^10^, ^11^, ^12^

With increasing evidence of the involvement of secondary mitochondrial dysfunction in congenital myopathy, the question arises as to whether the mitochondrial dysfunction often observed in congenital myopathies may include (seemingly) a primary impairment of individual OXPHOS enzyme function, which is a key feature of MD. This distinction is critical, as it has implications for diagnosis, patient classification and subsequent therapeutic strategies. Although sporadic reports and anecdotal evidence of OXPHOS complex dysfunction in congenital myopathies have been reported, the full extent of evidence has yet to be explored.

This systematic review aimed to assess the existing literature on patients with congenital myopathy and disease models in which mitochondrial dysfunction has been reported, with a particular focus on OXPHOS complex dysfunction; similar to what would be observed for a primary MD. By consolidating this evidence, we aim to improve the understanding of mitochondrial involvement in congenital myopathies and highlight potential diagnostic challenges. Recognizing the role of OXPHOS dysfunction in congenital myopathies could help prevent misdiagnoses and contribute to more targeted clinical management strategies for affected individuals.

## 2 METHODS

### 2.1 Study design

This is a descriptive and narrative systematic review aimed at summarizing the existing literature on the association between OXPHOS complex dysfunction and congenital myopathies.

The study was conducted using a structured search strategy in accordance with the Preferred Reporting Items for Systematic Reviews and Meta‐Analyses (PRISMA) guidelines.^13^, ^14^ The review was registered with PROSPERO, with registration number CRD420250652617, and approved by the North‐West University Health Research Ethics Committee (NWU‐HREC): NWU‐00109‐25‐A1.

### 2.2 Eligibility criteria

Studies were eligible for inclusion if they provided a genetically confirmed congenital myopathy disease model (in vivo and/or in vitro) that specifically reported on OXPHOS complex functionality using kinetic enzymatic activity assays, proteomics/protein and/or transcriptomics/RNA assays. Additional functional assays, such as respirometry and immunohistochemical or other histochemical staining (e.g. COX), were not included as eligibility criteria for the final selection but were considered supporting data when evaluating the final list of publications. Therefore, all articles published pre‐Sanger/Maxam‐Gilbert era (1977) were excluded due to the lack of molecular‐level accuracy required for this review.^3^, ^15^, ^16^ Furthermore, studies were excluded if: (1) the model was not genetically characterized, (2) lacked specific analysis of OXPHOS complex functionality (complex I‐V) and (3) the study was not an intervention of interest. The latter was defined as any study that investigated OXPHOS dysfunction outside the context of congenital myopathy (e.g. muscular dystrophy). Studies were additionally excluded under the category ‘does not meet study objective’ if they did not provide data linking OXPHOS dysfunction (whether mechanistically, functionally or transcriptionally) to a congenital myopathy‐causing gene or phenotype. This included studies that mentioned a congenital myopathy‐positive model but assessed unrelated pathways or failed to explore mitochondrial or OXPHOS‐related outcomes in the context of the myopathy.

In addition, due to the increased use of next‐generation sequencing methods, congenital myopathy is an evolving field with a continually expanding list of associated genes, particularly novel disease‐causing genes.^2^, ^17^ Therefore, a specific and isolated list of genes was not feasible to include, as it may inaccurately reflect the current development of the genetic landscape related to congenital myopathies. Ryanodine receptor type 1 (*RYR1*)‐related myopathy (*RYR1*‐RM) was included as a congenital myopathy subtype due to variants in this gene emerging as the leading cause of nondystrophic inherited neuromuscular disorders, specifically contributing to various congenital myopathies.^18^, ^19^, ^20^ No restrictions were imposed regarding model type, age, gender or ethnicity. Reviews, editorials, conference proceedings (posters) and personal opinions were not included to maintain the scientific rigour of this systematic review. Therefore, only indexed peer‐reviewed articles were considered, without inclusion of grey literature. However, referenced lists of reviews and other publications of interest were screened and where possible non‐English publications were translated with the aim of identifying further relevant studies for inclusion. Furthermore, in health‐related research, it is well‐established that excluding grey literature does not significantly influence the overall findings of related systematic reviews.^21^

### 2.3 Data sources

The literature search was completed using the following databases: PubMed, Scopus and Web of Science. These were accessed from 27 January 2025 to 30 January 2025, and a secondary search was completed on 8 March 2025 to ensure the inclusion of any newly published literature. The search was designed to include all fields (title, abstract, keywords, etc.) and no restrictions were made during this search. The search terms included a wide range of congenital myopathy and mitochondrial OXPHOS‐related terms: [(Congenital myopathy) OR (Nemaline myopathy) OR (Central core disease) OR (Central core myopathy) OR (Core myopathy) OR (Dusty core disease) OR (Bailey‐Bloch congenital myopathy) OR (Centronuclear myopathy) OR (Congenital fibre‐type 1 disproportion) OR (Congenital fibre‐type disproportion) OR (Core rod myopathy) OR (Multi‐minicore disease) OR (Myosin storage myopathy) OR (Native American myopathy) OR (Myotubular myopathy) OR (King Denborough) OR (RYR1‐related myopathy)] AND [(Mitochondri*) OR (Electron transport chain) OR (OXPHOS) OR (Oxidative phosphorylation) OR (Mitochondrial respiratory chain) OR (Mitochondrial complex)]. These search terms were used for the PubMed database, whereas other database searches utilized the search terms outlined in Data S1.

### 2.4 Data selection

All articles were retrieved and loaded into a single database using Covidence.^22^ The study selection process followed a two‐stage approach, namely, title and abstract screening, followed by a full‐text review. Two independent reviewers identified studies based on the predefined eligibility criteria. Any discrepancies between the reviewers were resolved through discussion; if consensus could not be reached, a third reviewer was consulted.

To assess the reporting quality of the studies after the selection process, Cohen's Kappa statistic was used alongside an adapted version of the Joanna Briggs Institute (JBI) critical appraisal tool, which incorporated a validated Likert scale,^23^ to provide a quantitative measurement of study quality. For this evaluation, we adapted specific questions from the JBI Case Reports Checklist, with a focus on enhancing the relevance of the quality assessment to this specific review. Two researchers independently evaluated the studies with the following questions:

1. Was the article published in a reputable scientific journal, as indicated by its ranking in the SCImago Journal Rank (SJR) system, which evaluates journal quality, reliability, and impact?
2. Was the patient's history clearly described and presented as a timeline? In the case of models, was the models' development clearly described?
3. Was the patient/disease model described using Human Phenotype Ontology terms or other clearly recognizable phenotypic characteristics?
4. Were the diagnostic tests/assessment methods and results clearly described or referenced to an acceptable publication?
5. Were the methods described for OXPHOS enzyme kinetics, omics or bioenergetic analyses clearly described or referenced to a publication that does so?
6. Were the results for OXPHOS enzyme kinetics, omics, or bioenergetic analyses clearly described that support the conclusions made by the authors?

Each question was rated as follows: ‘0 = no’, ‘1 = partly’ or ‘2 = yes’. Studies that answered all the questions with a total rating of ≥10 were classified as high quality, studies rating between 5 and 9 were considered intermediate quality, whereas those studies with a rating of ≤4 were classified as low quality. Instead of applying a fixed SJR cut‐off, we used the publishing journal's SJR quartile (Q): studies published in Q1 journals were rated as high quality, those in Q2 and Q3 as intermediate, and those in Q4 or unranked journals as low quality. While higher SJR scores may favour positive results, this criterion was balanced by including all study types regardless of outcome. Importantly, to mitigate potential publication bias, no publications were excluded based on their quality score; rather, the scoring system was used to gauge overall methodological rigour and inform the interpretation of findings within the context of study quality.

## 3 RESULTS

### 3.1 Study characteristics

**FIGURE 1:**
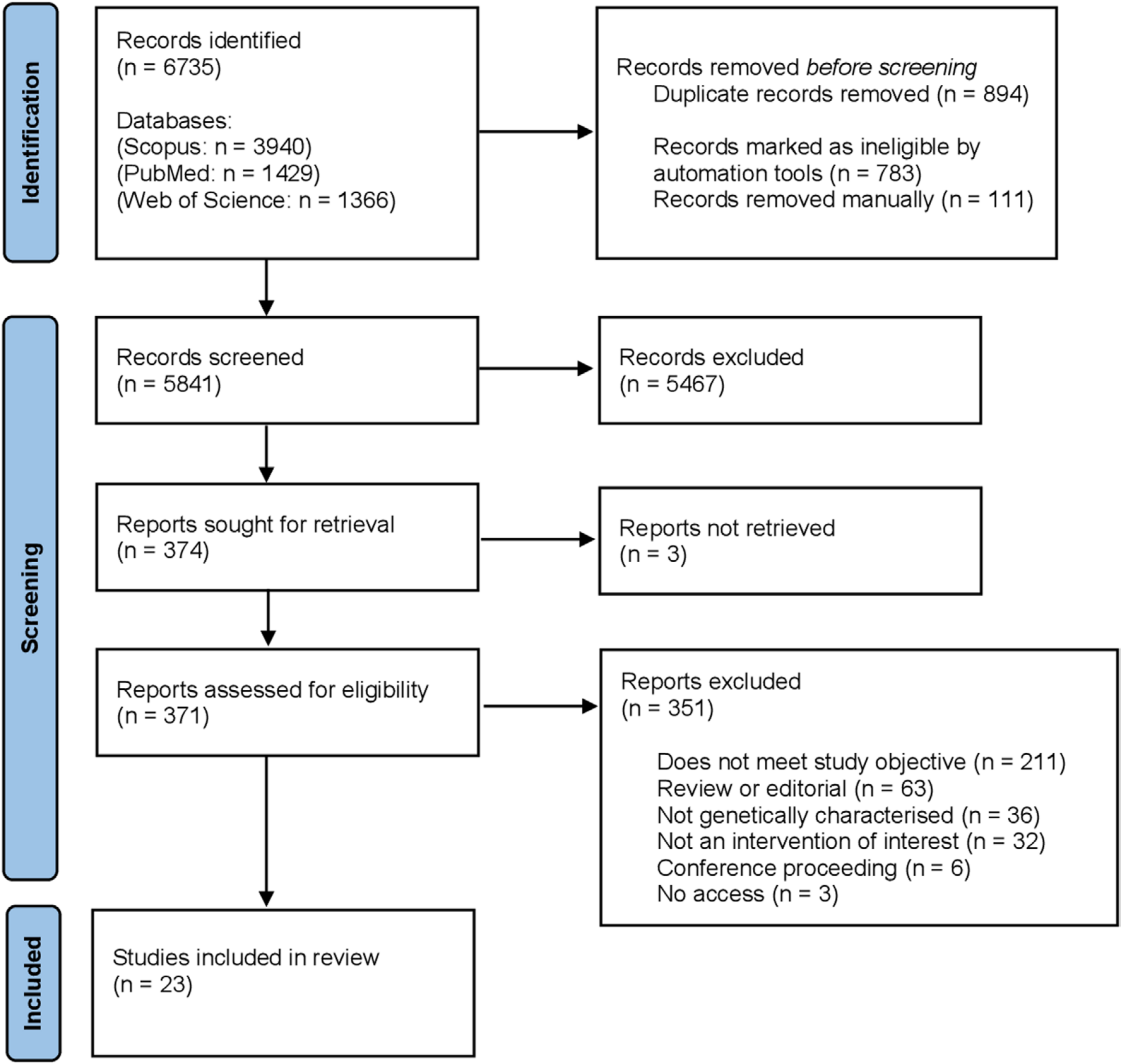
Preferred reporting items for systematic reviews and meta‐analyses (PRISMA) flow diagram for results of search strategy.

As depicted in Figure 1, *n* = 6735 studies were retrieved from the relevant databases. Thereafter, duplicates (*n* = 894) were removed, resulting in *n* = 5841 studies, which underwent title and abstract screening. Of these, *n* = 5467 were excluded as irrelevant. This resulted in *n* = 347 studies that were sought for full‐text analysis. Three studies were not retrieved and, consequently, only *n* = 371 studies were fully assessed. Following this, a further *n* = 351 studies were excluded for the reasons listed in Figure 1, resulting in a total exclusion of 99.6% of the publications. Ultimately, 23 studies (published between 2009 and 2025) were included in this review. Based on the finalized selection of articles, data extraction included: (1) congenital myopathy cases (disease model/patient/cell line), (2) diagnosed congenital myopathy type, (3) genetic variant, (4) summarized clinical symptoms/presentation and (5) OXPHOS complex activity with the relevant functional assay completed. The data were organized into a series of tables, which are referenced later in this article.

A total of six studies investigated isolated patients with congenital myopathy, whereas one study combined both patients and a cell culture model. Twelve studies used isolated mouse models, and two additional studies used mouse models in conjunction with cell culture models. Two final studies reported the use of isolated cell culture models. Thus, a total of 45 study cases were included (hereafter referred to as ‘cases’, encompassing patients, cell lines and animal models from the respective studies).

### 3.2 Quality assessment of the included studies

Cohen's Kappa statistic for agreement between the two raters on the quality classification was 0.74, indicating substantial agreement.^24^ Approximately 80% of the studies were classified as high quality, and 20% were considered intermediate quality by both raters. No studies were rated as low quality, indicating a generally high standard of methodological quality across the included studies (Tables S1.1–1.2). Of the 23 publications reviewed, two received a total score of 2 for each question, resulting in an overall quality criteria score of 12. The majority of studies (*n* = 21) were published in Q1 journals, indicating a high ranking of publication sources. Patients' history or rationale for model development was clearly described and presented in four studies, whereas this information was either partially mentioned, unclear or absent from the remaining studies. Furthermore, nine studies used HPO terms or recognizable phenotypical characteristics to describe the patient/disease model. Diagnostic tests or evaluations of the patient/disease model, together with the results, were clearly outlined in most of the studies (*n* = 22). The OXPHOS results of interest were clearly described and supported the conclusion of the respective studies in 22 publications, whereas the methods used to determine OXPHOS function were relevant and explicitly described or referenced in 20 publications.

### 3.3 Congenital myopathy cases with associated OXPHOS complex dysfunction

In this review, we screened and examined 23 publications of genetically confirmed congenital myopathy cases with reported deficiencies of isolated and/or combined OXPHOS complexes (or subunits) (Table 1) that would be consistent with a positive biochemical diagnosis of an MD. Unlike traditional histochemical staining used to identify mitochondrial dysfunction in muscle—which generally involves end‐point assays—these publications specifically reported normalized, kinetic single enzyme assays in muscle (or other relevant affected tissues or cell lines) and/or quantitative RNA transcript/protein analyses to infer a dysfunction similar to that expected in inherited OXPHOS dysfunction. Respirometry data were also included as supporting data. From the 23 publications and 45 cases (including 23 human and 17 mice tissue samples, and five cell line models) investigated, 18 cases of congenital myopathy reported on enzyme kinetics. Of these, 15 (including all human cases) reported an enzymatic dysfunction of one or a combination of respiratory chain complexes, with the absence of significant changes reported in three cases. Notably, complex I and/or complex IV deficiencies were found in the vast majority (13/15) of these cases, which is consistent with the relative prevalence of OXPHOS enzyme dysfunctions reported for MD in humans.

**TABLE 1:** Reported CM cases with OXPHOS complex variability.

Patient/genetic model^a^	Genetic variants^b^	OXPHOS‐related analyses^c^			
		Enzymology	Protein/*Transcripts* (subunits)	Respirometry	References
P1: F (18y)	*SCN4A*:(p.Arg1454Trp; p.Asn1205Lys)	CI & CI + CIII ↓			^35^
P2‐P9: M (8x; 11y‐39y)	*RYR1*:p.Arg2336His; p.Thr1431=; p.Val4234Leu; p.Val2627Met; p.Ser1728Phe; p.Asp2431Val; p.Gly4734Glu; p.Thr2206Met		*CI (various subunits) ↓*		^28^
P10: M (7 m)	*MTM1*:p.Trp499*	CI ↓, CII ↓, CIII & CIV ↔, CII + CIII ↓; CI‐CIV ↔, CII + CIII ↔ (liver)			^36^
P11: M (11 m)	*MTM1*:p.Arg421Gln	CI ↓, CII & CIII ↔, CIV ↓, CII + CIII ↔			^36^
P12	*ACTA1*:p.Asn254His	CI ↓, CII‐CIV ↔	CI (NDUFB8) ↔, CIV (COXI) ↔		^37^
P13	*STAC3*:p.Trp284Ser	CI & CII ↔, CIII ↓, CIV & CII + CIII ↔			^38^
P14	*RYR1*:(p.Ile2781Argfs*49; p.His3976Tyr)	CI‐CIII ↔, CIV & CII + CIII ↓			^38^
P15	*RYR1:(*p,Val482Met; c.11193 + 1G>A^d^)	CI ↓, CII‐CIV & CII + CIII ↔			^38^
P16: M (10y)	*RYR1*:(p.Gly4935Valfs*2; p.Arg3539His)	CI‐CIV ↓			^39^
P17: F (6y)	*RYR1*:(p.Asn4575Thr)		CII (SDHB) ↑, CIII (UQCRC2) ↑		^40^
P18: M (7y)	*RYR1*:(p.Ile1571Val; p.Leu3136Argfs*75)		CV (ATP5A) ↑		^40^
A1: Mouse	*Ryr1* ^G2435R/G2435R^		*CI (NDUFS1, NDUFS2) ↓, CII (SDHA) ↓, CIII (UQCRC2) ↓*	CIV ↓; CI, CI + CII ↑; Leak, ETS ↔	^25^
A2: Mouse	*Ryr1* ^G2435R/G2435R^		*↔*	↔	^25^
A3: Mouse	*Ryr1* ^T4826I/+^		*CIII (UQCRC2) ↓, CV (ATP5A) ↓*	CIV ↔, CI, CI + CII ↓; CI + CII ↑, Leak ↓	^25^
A4: Mouse	*Dnm2* ^*R369W/+*^		CI (NDUFB8) ↑, CII (SDHB) ↑, CIII (UQCRC2) ↑, CIV (MT‐CO1) ↑, CV (ATP5A) ↑		^41^
A5: Mouse	*Ryr1* Q1970fs*16/A4329D		CI (NDUFV3) ↓, CIII (UQCRB) ↓		^42^
A6: Mouse	*Sepn1* ^−/−^	CI ↓(df, qd, ta); CI ↔ (edl)		CI, CII, CI + CII, CIV ↓	^43^
A7: Mouse	*Sepn1* ^−/−^		CI (NDUFB8), CII (SDHB), CIII (UQCRC2), CIV (MT‐CO1), CV (ATP5A) ↔		^44^
A8: Mouse	*Ryr1* ^A163C^	CI, CIII, CIV ↓; CII, CV ↔		P/O ↔; RCR, State 3 ↓	^45^
A9: Mouse	*Ryr1* ^Y522S/+^		CIV (COX IV) ↑		^46^
A10: Mouse	*Mtm1* ^−/−^		CI (NDUFA3), CIII (MT‐CYB), ↓; CV (ATP51C) ↑	CIV ↓	^26^
A11: Mouse	*Mtm1* ^‐/Y^		*OXPHOS ↓ (unspecified)*		^47^
A12: Mouse	*Neb* cKO	CI‐CV ↔	CI ‐ CV ↑ (various subunits)	RCI ↓	^48^
A13: Mouse	*HAS‐Cre; Dnm2* ^*fl/fl*^		CI (NDUFA9), CII (SDHA) ↓		^49^
A14: Mouse	Tg(*ACTA1* ^*D286G*^)	CI‐CV ↔	CI, CII, CIV ↑ (various subunits)	RCI ↔	^27^
A15: Mouse	*ACTA1* ^*H40Y*^	CI‐CV ↔	CI ↑↓; CIV ↑, CV ↓ (various subunits)	RCI ↓	^27^
A16: Mouse	*MCK‐Cre* ^+^; *Speg* ^*fl/fl*^		CI (NDUFB8), CII (SDHB), CIII (UQCRC2), CIV (MT‐CO1), CV (ATP5A) ↔ CI (NDUFA5), CIII (UQCR10), CV (ATP5MK) ↑		^50^
A17: Mouse	*Mtm1* ^*‐/Y*^	CIV ↓			^51^
C(P)1: Fibroblasts	*ACTA1*:p.Val45Phe		CI (NDUFA9, NDUFS1, MT‐ND1), CII (SDHB), CIII (UQCRC2), CIV (MT‐COX2, COXIV), CV (ATP5F1A) ↓	Basal, Maximal, Spare respiratory capacity, ATP production ↓	^52^
C(P)2: Fibroblasts	*ACTA1*:p.Asn254Tyr				^52^
C(P)3: Fibroblasts	*NEB*:(p.Thr3441Pro; pArg4557*)				^52^
C(P)4: Fibroblasts	*NEB*:(p.Leu8137Phefs*18; p.Arg2809*)				^52^
C(P)5: Myocytes & Myoblasts	*Mtm1* pathogenic variants	CIV ↓			^51^
C1: HeLa	*Sepn1 KO*	CII, CIV ↓			^43^
C2: C2C12	*Ryr1* KD		CI (NDUF8), CII (SDHB), CIII (UQCRC2), CV (ATP5A) ↑	Basal ↑	^40^
C3: C2C12	*Ryr1* KD		CI, CII, CIII, CV ↑ (various subunits)	State 2 & 3 ↓	^53^
C4: C2C12	*Mtm1* KD	CIV ↓			^51^
C5: C2C12	*Sepn1* KO	CII ↓; CIV ↓			^43^

*Note*: Clinical and other information of cases, as well as additional information of genetic variants, is provided in Table S1.

Abbreviations: ↑, increased activity/upregulated; ↓, dysfunction/downregulated; ↔, no change; CI–V, complex I–V; df, diaphragm; edl, extensor digitorum longus; KD, siRNA knock down; KI, knock in; KO, knock out; Open spaces, no analyses done; P/O, phosphate/oxygen ratio; qd, quadriceps; RCI, respiratory control index; RCR, respiratory control ratio; ta, tibialis anterior.

^a^ Arbitrary number for a list of 45 patients (P), mouse (A) or cell lines (C) is given. For patients, the sex, number of cases (x), and age of onset in years (y) or months (m) are given, where known.

^b^ Variants in brackets are heterozygous/compound heterozygous; all others are homozygous.

^c^ All analyses are in skeletal muscle, unless indicated otherwise or the muscle is specified due to variation.

^d^ Intronic variant.

When analysing those studies in which quantitative transcript or protein analyses of the OXPHOS complex subunits were performed, a significant reduction in expression was reported in 20/30 (66.7%) cases, often coinciding with reduced respiration. An increase in expression was observed in the remaining 10 cases (33.3%), even though functional respirometry analyses in three of these cases indicated an OXPHOS dysfunction. Two cases presented with variable results in complex V subunits,^26^, ^27^ whereas one case reported no variability.^25^

A comparison of the genes and congenital myopathy subtypes will be described in the following sections, but upon closer examination, there does not appear to be a definitive relationship between the OXPHOS complexes involved and the genes affected. This may be seen for *RYR1—*the most frequently affected protein in the reported cases—which can result in several congenital myopathy phenotypes,^18^ with all five of the complexes variably involved across the cases described.

### 3.4 Congenital myopathy‐associated genes resulting in OXPHOS complex dysfunction

**FIGURE 2:**
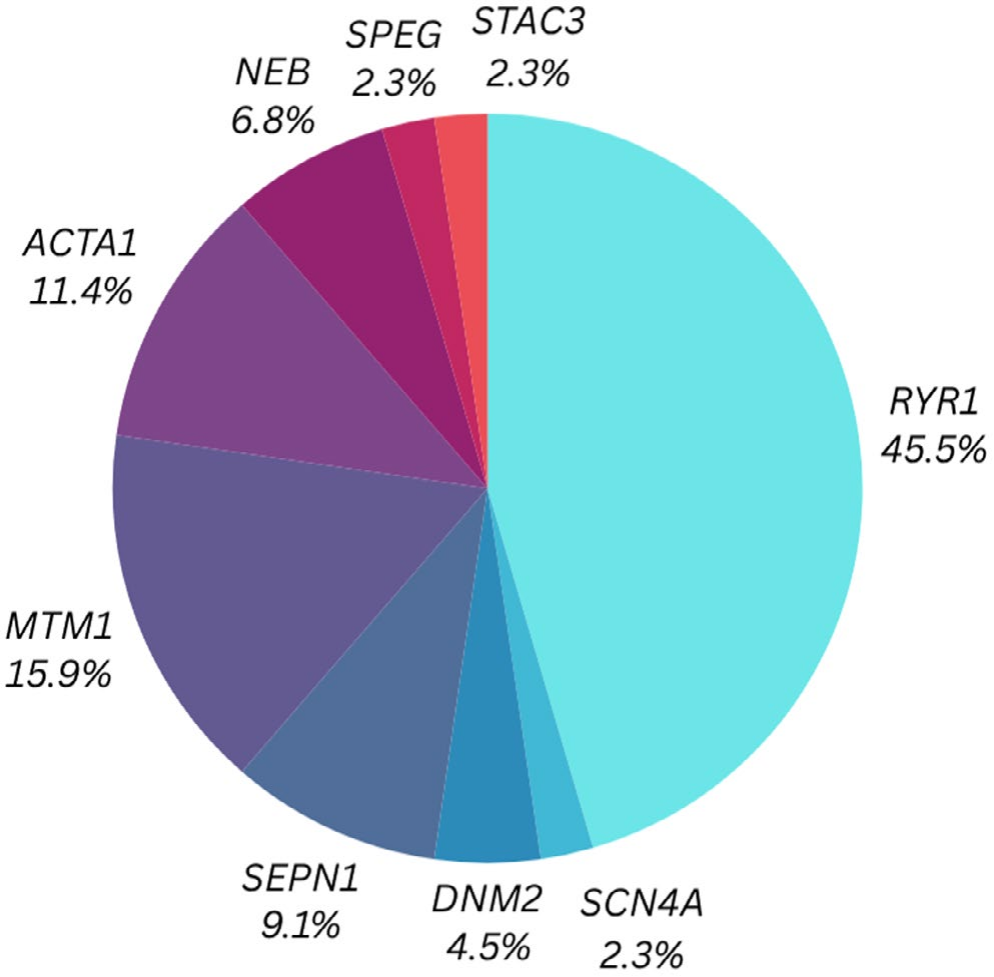
Congenital myopathy‐associated genes in publications in which OXPHOS complex variation was reported.

Evaluation of the 44 cases for which genetic confirmation was available revealed a total of nine congenital myopathy‐associated genes with pathogenic variants (Figure 2). Only 44 cases were included here, since one of the eight patients reported by Chang et al.^28^ had an unknown genetic variant. Figure 2 represents a simplified overview highlighting the key genes involved in the publication set, with a summary of the genetic variants represented in Table 1 (also see Table S3). A condensed overview of the specific protein functions is listed in Table 2.

**TABLE 2:** Key proteins identified in the congenital myopathy cases with their general function.

Protein	UniProt ID	Muscle function	Molecular function	Trigger
RYR1 (Ryanodine receptor 1)	P21817	Mediates Ca^2+^ release from SR to the cytosol, essential for muscle contraction during ECC	Calcium release channel	Activated by depolarization of T‐tubules via DHPR (Cav1.1)
SCN4A (Sodium channel protein type 4 subunit alpha)	P35499	Initiates action potential in excitable membranes, essential for muscle contraction	Voltage‐gated sodium channel	Triggered by membrane depolarization
DNM2 (Dynamin 2)	P50570	Involved in membrane organization, autophagy, and actin cytoskeleton remodelling; additionally involved in muscle vesicular trafficking	GTPase regulating endocytosis	Activated via lipid binding and oligomerization; enhances GTPase activity during self‐assembly
SEPN1 (Selenoprotein N)	Q9NZV5	Maintains redox balance and Ca^2+^ homeostasis in muscle ER; linked to muscle integrity, stress resistance, and efficient ECC.	Redox‐activated ER transmembrane selenoprotein with reductase activity. Modulates RYR activity.	Responds to oxidative or ER stress; restores calcium pump ATP2A2 activity
MTM1 (Myotubularin 1)	Q13496	Stabilizes MTMR12 levels, regulates muscle maintenance and mitochondrial morphology and positioning	Lipid phosphatase that dephosphorylates PI3P and PI(3,5)P_2_	Constitutively active; specifically, function is regulated via membrane binding and protein interactions^54^
ACTA1 (Actin, Alpha Skeletal Muscle)	P68133	Forms the core of thin filaments in sarcomeres, required for contraction and structural integrity^55^	Structural actin protein involved in cell motility and cytoskeletal maintenance	Contraction driven by myosin heads and actin filaments cross‐bridge formation^56^
NEB (Nebulin)	P20929	Maintains structural integrity of sarcomeres and associated membrane systems in muscle fibres	Giant structural protein that binds and stabilizes F‐actin	Constitutive structural role; no signal‐based trigger^57^
SPEG (Striated Muscle Enriched Protein Kinase)	Q15772	Supports the triad structure and ECC in skeletal and cardiac muscle^58^	Muscle‐specific kinase involved in ECC and membrane stabilization^58^	Developmentally regulated; may be modulated by Ca^2+^ signalling as well as muscle differentiation signals^59^
STAC3 (SH3 and Cysteine‐Rich Domain 3)	Q96MF2	Essential for ECC in skeletal muscle; supports normal Ca^2+^ release and contraction	Scaffold/adaptor protein that enhances *CACNA1S* expression and activity	Functions during T‐tubule depolarization

*Note*: Data were compiled from UniProtKB entries.^60^ Human Swiss‐Prot IDs are listed and paraphrased for clarity; additional references were included within the table when required.

Abbreviations: ATP2A2, sarcoplasmic/endoplasmic reticulum calcium ATPase 2; *CACNA1S*, calcium voltage‐gated channel subunit alpha1 S gene; Ca^2+^, calcium ion; Cav1.1, calcium voltage‐gated channel subunit alpha1 S protein; DHPR, dihydropyridine receptor; ECC, excitation–contraction coupling; ER, endoplasmic reticulum; F‐actin, filamentous actin; GTPase, guanosine triphosphatase; MTMR12, myotubularin‐related protein 12; PI(3,5)P₂, phosphatidylinositol 3,5‐bisphosphate; PI3P, phosphatidylinositol 3‐phosphate; SR, sarcoplasmic reticulum.

Of the 23 publications included in this systematic review, variants in nine congenital myopathy‐associated genes were found to contribute to OXPHOS variation, with 46% of cases presenting with variants in the *RYR1* gene. This is consistent with the relatively high reported occurrence of *RYR1* variants in congenital myopathy.^19^, ^20^ Genetic variants in *MTM1* and *ACTA1* accounted for a further 16% and 11% of the cases in this review, respectively. Subsequently, variants in *SEPN1*, *NEB* and *DNM2* accounted for 9%, 7% and 5% of the cases, respectively. Lastly, variants in *STAC3, SCN4A* and *SPEG* were only presented once (2%, respectively) within the cases. To compare the functional variation of the nine genes identified, Table 2 lists the general protein function of each. Considering this as well as the vital roles of these proteins in skeletal muscle function, their contribution to excitation–contraction coupling (ECC), sarcomere stability, muscle repair and membrane integrity is evident. Interestingly, all the proteins are involved in calcium regulation or calcium‐dependent processes, whether by mediating the downstream response to calcium, the direct regulation of calcium, or calcium‐dependent contractile properties. Thus, these proteins may contribute to defects in energy production and metabolic stability, as mitochondrial activity is highly sensitive to calcium fluctuations.^29^

### 3.5 Variability in congenital myopathy subtypes with OXPHOS complex variability

Congenital myopathy represents a diverse group of inherited muscular disorders characterized by a variety of phenotypes, with patients commonly presenting both structural abnormalities and impaired muscle function. Congenital myopathies encompass multiple main subtypes, including, but not limited to, core myopathy, nemaline myopathy, centronuclear myopathy, congenital fibre‐type disproportion myopathy and myosin storage myopathy. Each of these can be further subdivided into specific subgroups based on genetic, histological and clinical criteria.^30^

**FIGURE 3:**
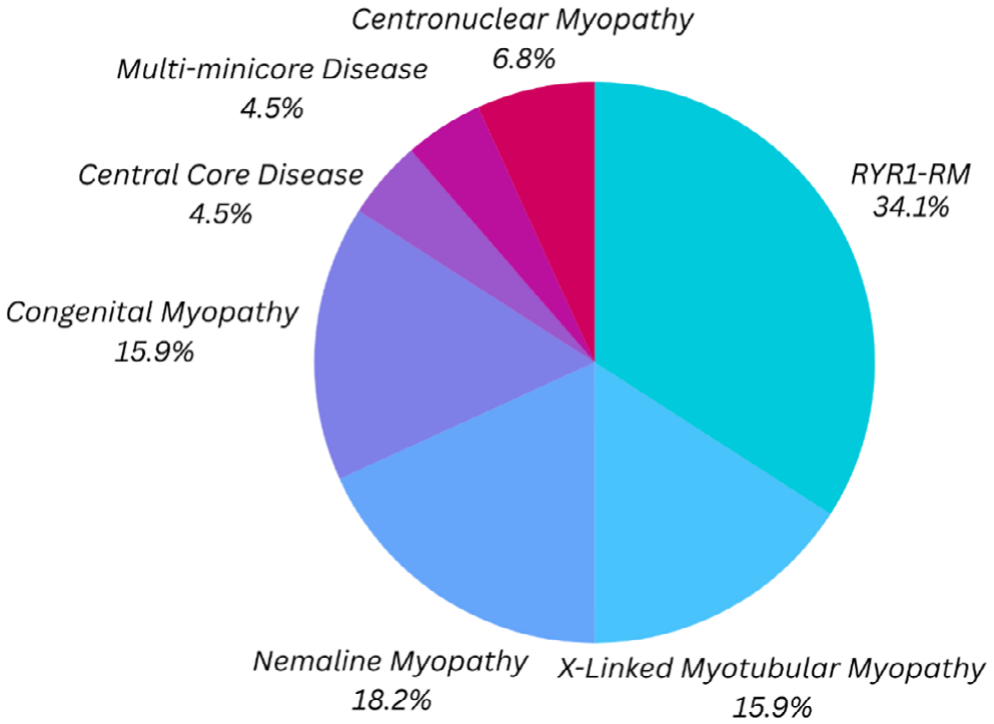
Congenital myopathy subtypes in publications in which OXPHOS complex variation was reported. RYR1‐RM, RYR1‐related myopathy.

A total of 44 finalized cases (excluding one case reported by Chang et al.^28^) and their corresponding congenital myopathy diagnoses are presented in Figure 3. When a specific classification (subtype or subgroup) was not stipulated, the study case was classified under the general term congenital myopathy. An exception was made for cases involving *RYR1* variants, which were specifically labelled as *RYR1*‐RM unless otherwise stated. The specific clinical phenotypes and other diagnostic data for each of the cases are listed in Table S3.

As depicted in Figure 3 and corresponding to Figure 2, *RYR1*‐RM accounts for the majority of cases within this study group, whereas other gene‐related myopathies (labelled here as congenital myopathy) account for an additional 16%. Further classification (if possible, in the models) would be required to accurately categorize these cases. In addition, classified by the presence of nemaline rods, nemaline myopathy is regarded as the more common form of congenital myopathy^31^; therefore, it is of no surprise that within our study group, this myopathy type represented a further 18% of cases, making it the second most represented group. Centronuclear myopathy, together with its subgroup X‐linked myotubular myopathy (often referenced interchangeably as X‐linked centronuclear myopathy), represented a large section of cases, followed by two core myopathy subgroups: central core disease and multi‐minicore disease. Therefore, four of the six (including *RYR1*‐RM) commonly classified congenital myopathy subtypes are represented in our study group.

Moreover, similar to the variability in genes resulting in the secondary OXPHOS variation, it is evident that this observation is not limited to one specific subtype of congenital myopathy, but rather to a wider diversity of congenital myopathy subtypes. This finding suggests that the diagnostic histological features observed in congenital myopathies with OXPHOS variation may not represent distinct pathophysiological processes but rather reflect overlapping or shared mechanisms. As the gene functions would suggest, it may correlate with aberrant calcium modulation and its cellular consequences.

## 4 DISCUSSION

Our review summarizes the current evidence linking congenital myopathy with secondary mitochondrial OXPHOS dysfunction, specifically highlighting the occurrence of OXPHOS complex impairment. As previously mentioned, our systematic review identified 23 publications encompassing 45 critically evaluable cases, of which 35/45 (78%) presented with an OXPHOS complex dysfunction, supporting the assertion that OXPHOS complex dysfunction is of high prevalence in congenital myopathies. However, despite this observation, we were unable to establish any genotype–phenotype relationship between the genes, congenital myopathy subtypes and/or specific OXPHOS complex dysfunction in the identified cases; neither was the nature of the dysfunction isolated to a single gene, subtype or complex. Such an observation may indicate that secondary OXPHOS dysfunction in congenital myopathy arises due to a broader, secondary co‐dysregulation of the mitochondrial OXPHOS subunits. Considering the common role of these congenital myopathy‐associated proteins in calcium modulation, it is plausible that calcium‐mediated signalling may be the key retrograde signalling pathway that results in OXPHOS dysregulation.

Our review presents evidence that secondary mitochondrial OXPHOS dysfunction in cases of congenital myopathy can arise from non‐MD‐associated genetic variants. These findings reinforce the conceptual framework that multiple mechanisms, including transcriptional dysregulation, post‐translational modifications, and downstream structural impairments, may contribute to OXPHOS dysfunction, rather than a sole direct enzymatic defect. The identification of calcium metabolism‐related genes in the included studies suggests calcium‐mediated retrograde signalling is likely involved in OXPHOS gene regulation.^32^ However, the observed reduction in OXPHOS subunit protein expression in most of the studies reporting expression data does not exclude post‐translational mechanisms, such as protein misfolding, degradation or oxidative damage. In particular, dysregulation of intracellular calcium buffering may impair mitochondrial calcium homeostasis, thereby promoting increased ROS production, oxidative stress and subsequent secondary impairment of OXPHOS complexes.

This multilayered regulatory framework aligns with previous literature describing how calcium‐dependent signalling pathways influence OXPHOS via transcriptional co‐activators (such as PGC‐1α and its partners ERRA, NRF1 and NRF2) as well as mitochondrial transcription factors (TFAM, TFB1M and TFB2M).^33^, ^34^ However, continued in‐depth investigations into these pathways would be critical to understanding the specific mechanisms underlying secondary OXPHOS dysfunction in congenital myopathy.

## 5 CONCLUSION

In conclusion, previous sporadic scientific and anecdotal reports have described dysfunction of the OXPHOS complexes in patients with suspected congenital myopathy. However, there is currently no consensus regarding the findings of these studies. This lack of clarity prompted the current systematic review to determine whether such observations were more common than previously recognized. The main finding presented in this review is that OXPHOS complex dysfunction in congenital myopathies may indeed be part of the pathophysiology as could be observed if quantitative analyses of the OXPHOS complexes are performed more regularly. It challenges the recognition of OXPHOS complex dysfunction as a specific diagnostic hallmark of MD and, with overlapping clinical features and mitochondrial involvement, underscores the importance of clinical and genetic evaluations for MD and congenital myopathy.

## AUTHOR CONTRIBUTIONS

Megan J. du Preez: designed study; collected and analysed data; independent reviewer 1; wrote paper; Maryke Schoonen: analysed data; moderating reviewer; wrote paper; Monray E. Williams: analysed data; wrote paper; Michelle Bisschoff: analysed data; wrote paper; Francois H. van der Westhuizen: conceptualized study; designed study; analysed data; independent reviewer 2; wrote paper.

## CONFLICT OF INTEREST STATEMENT

The authors declare no conflicts of interest.

## Supporting information


**Data S1.** Search terms/strategy.

**Table S3.** Genetic variants, subtypes, and associated symptoms of congenital myopathy cases and models.

## Data Availability

Data sharing is not applicable to this article as no datasets were generated or analysed during this study.
