# Supplementary material for: OXPHOS complex deficiency in congenital myopathy: A systematic review
Source: Eur J Clin Invest. 2025 Sep 11;55(11):e70114. doi: 10.1111/eci.70114 (PMC12517249; doi:10.1111/eci.70114)
Supplement: Supplementary file 1 — Data S1. Search terms/strategy. [file ECI-55-e70114-s002.docx]

**Supplementary Material 1:**

**Search Terms/Strategy:**

**PubMed:**

((Congenital myopathy) OR (Nemaline myopathy) OR (Central core disease) OR (Central core myopathy) OR (Core myopathy) OR (Dusty core disease) OR (Bailey-Bloch congenital myopathy) OR (Centronuclear Myopathy) OR (Congenital Fibre type 1 disproportion) OR (Congenital Fibre-type disproportion) OR (Core rod myopathy) OR (Multi-minicore disease) OR (Myosin storage myopathy) OR (Native American Myopathy) OR (Myotubular myopathy) OR (king denborough) OR (RyR1-related myopathy)) AND ((Mitochondri*) OR (Electron transport chain) OR (OXPHOS) OR (Oxidative phosphorylation) OR (Mitochondrial respiratory chain) OR (Mitochondrial complex))

**Scopus:**

( "Congenital myopathy" OR "Nemaline myopathy" OR "Central core disease" OR "Central core myopathy" OR "Core myopathy" OR "Dusty core disease" OR "Bailey-Bloch congenital myopathy" OR "Centronuclear Myopathy" OR "Congenital Fibre type 1 disproportion" OR "Congenital Fibre-type disproportion" OR "Core rod myopathy" OR "Multi-minicore disease" OR "Myosin storage myopathy" OR "Native American Myopathy" OR "Myotubular myopathy" OR "king denborough" OR "RyR1-related myopathy" ) AND ( "(Mitochondri*)" OR "Electron transport chain" OR "OXPHOS" OR "Oxidative phosphorylation" OR "Mitochondrial respiratory chain" OR "Mitochondrial complex" )

**Web of Science:**

((Congenital myopathy) OR (Nemaline myopathy) OR (Central core disease) OR (Central core myopathy) OR (Core myopathy) OR (Dusty core disease) OR (Bailey-Bloch congenital myopathy) OR (Centronuclear Myopathy) OR (Congenital Fibre type 1 disproportion) OR (Congenital Fibre-type disproportion) OR (Core rod myopathy) OR (Multi-minicore disease) OR (Myosin storage myopathy) OR (Native American Myopathy) OR (Myotubular myopathy) OR (king denborough) OR (RyR1-related myopathy)) AND ((Mitochondri*) OR (Electron transport chain) OR (OXPHOS) OR (Oxidative phosphorylation) OR (Mitochondrial respiratory chain) OR (Mitochondrial complex))

**Supplementary Tables:**

**Supplement Table 1.1: Quality assessment (Independent researcher 1)**

|  | **Assessment scores** | | | | | | | **Quality classification** |
| --- | --- | --- | --- | --- | --- | --- | --- | --- |
| **Reference** | **Q1** | **Q2** | **Q3** | **Q4** | **Q5** | **Q6** | **Total** |  |
| 35 | 1 | 2 | 2 | 2 | 0 | 2 | 9 | Intermediate |
| 25 | 2 | 1 | 1 | 2 | 2 | 2 | 10 | High |
| 28 | 2 | 0 | 1 | 2 | 2 | 2 | 9 | Intermediate |
| 41 | 2 | 1 | 2 | 2 | 2 | 2 | 11 | High |
| 42 | 2 | 1 | 1 | 2 | 2 | 2 | 10 | High |
| 43 | 2 | 1 | 2 | 2 | 2 | 2 | 11 | High |
| 44 | 2 | 2 | 2 | 2 | 2 | 2 | 12 | High |
| 45 | 2 | 2 | 2 | 2 | 2 | 2 | 12 | High |
| 36 | 1 | 1 | 2 | 2 | 2 | 2 | 10 | High |
| 52 | 2 | 0 | 1 | 2 | 2 | 2 | 9 | Intermediate |
| 37 | 2 | 2 | 2 | 2 | 0 | 2 | 10 | High |
| 46 | 2 | 1 | 2 | 2 | 2 | 2 | 11 | High |
| 26 | 2 | 1 | 2 | 2 | 2 | 2 | 11 | High |
| 38 | 2 | 0 | 1 | 2 | 2 | 2 | 9 | Intermediate |
| 47 | 2 | 1 | 1 | 2 | 2 | 2 | 10 | High |
| 48 | 2 | 1 | 1 | 2 | 2 | 2 | 10 | High |
| 40 | 2 | 0 | 0 | 2 | 2 | 2 | 8 | Intermediate |
| 49 | 2 | 1 | 1 | 2 | 2 | 2 | 10 | High |
| 27 | 2 | 1 | 1 | 2 | 2 | 2 | 10 | High |
| 53 | 2 | 1 | 1 | 2 | 2 | 2 | 10 | High |
| 50 | 2 | 1 | 0 | 2 | 2 | 2 | 9 | Intermediate |
| 51 | 2 | 1 | 2 | 2 | 2 | 2 | 11 | High |
| 39 | 2 | 2 | 2 | 2 | 2 | 2 | 12 | High |

**Supplement Table 1.2: Quality assessment (Independent researcher 2)**

|  | **Assessment scores** | | | | | | | **Quality classification** |
| --- | --- | --- | --- | --- | --- | --- | --- | --- |
| **Reference** | **Q1** | **Q2** | **Q3** | **Q4** | **Q5** | **Q6** | **Total** |  |
| 35 | 1 | 2 | 2 | 2 | 0 | 1 | 8 | Intermediate |
| 25 | 2 | 1 | 1 | 2 | 2 | 2 | 10 | High |
| 28 | 2 | 0 | 0 | 2 | 2 | 2 | 8 | Intermediate |
| 41 | 2 | 2 | 2 | 2 | 2 | 2 | 12 | High |
| 42 | 2 | 2 | 2 | 2 | 2 | 2 | 12 | High |
| 43 | 2 | 2 | 2 | 2 | 2 | 2 | 12 | High |
| 44 | 2 | 1 | 1 | 2 | 2 | 2 | 10 | High |
| 45 | 2 | 2 | 2 | 2 | 2 | 2 | 12 | High |
| 36 | 1 | 2 | 2 | 2 | 2 | 2 | 11 | High |
| 52 | 2 | 1 | 1 | 2 | 2 | 2 | 10 | High |
| 37 | 2 | 2 | 2 | 2 | 0 | 2 | 10 | High |
| 46 | 2 | 2 | 2 | 2 | 2 | 2 | 12 | High |
| 26 | 2 | 2 | 2 | 2 | 2 | 2 | 12 | High |
| 38 | 2 | 0 | 1 | 2 | 2 | 2 | 9 | Intermediate |
| 47 | 2 | 1 | 2 | 2 | 2 | 2 | 11 | High |
| 48 | 2 | 2 | 2 | 1 | 1 | 2 | 10 | High |
| 40 | 2 | 0 | 0 | 2 | 2 | 2 | 8 | Intermediate |
| 49 | 2 | 1 | 2 | 2 | 2 | 2 | 11 | High |
| 27 | 2 | 2 | 2 | 2 | 2 | 2 | 12 | High |
| 53 | 2 | 1 | 1 | 2 | 2 | 2 | 10 | High |
| 50 | 2 | 1 | 1 | 2 | 2 | 2 | 10 | High |
| 51 | 2 | 1 | 1 | 2 | 2 | 2 | 10 | High |
| 39 | 2 | 2 | 2 | 2 | 2 | 2 | 12 | High |
